# Activation of Subthalamic Nucleus Stop Circuit Disrupts Cognitive Performance

**DOI:** 10.1523/ENEURO.0159-20.2020

**Published:** 2020-10-07

**Authors:** Jonathan Heston, Alexander Friedman, Mustafa Baqai, Nicholas Bavafa, Adam R. Aron, Thomas S. Hnasko

**Affiliations:** 1 Department of Neurosciences, University of California, San Diego, La Jolla, CA 92093; 2 Department of Psychology, University of California, San Diego, La Jolla, CA 92093; 3 Research Service, Veterans Affairs San Diego Healthcare System, San Diego, CA 92161

**Keywords:** basal ganglia, cognition, interruption, mice, stopping, subthalamic nucleus

## Abstract

Much evidence supports a fundamental role for the subthalamic nucleus (STN) in rapidly stopping behavior when a stop signal or surprising event occurs, but the extent to which the STN may be involved in stopping cognitive processes is less clear. Here, we used an optogenetic approach to control STN activity in a delayed-match-to-position (DMTP) task where mice had to recall a response location after a delay. We first demonstrated that a surprising event impaired performance by both slowing the latency to respond and increasing the rate of errors. We next showed that these effects could be mimicked by brief optogenetic activation of the STN. Further, inhibiting STN during surprise blocked surprise-induced slowing, although without changing surprise-induced errors. These data are consistent with the hypothesis that STN is recruited by surprise to slow responding and that this can also interrupt cognitive processes. Under normal conditions STN-mediated stopping of behavior may slow or stop ongoing cognition to facilitate cognitive reorienting and adaptive responses to unexpected sensory information, but when malfunctioning, it could produce pathologies related to over-rigidity or increased distractibility.

## Significance Statement

While a central role for subthalamic nucleus (STN) in slowing and stopping behavior is well established, recent studies in human subjects have expanded this idea and suggest that STN-related circuits that stop behavior can also stop cognitive processes, i.e., thought. To test this, we developed a cognitive task in mice. We show that surprising sensory stimuli and optogenetic STN activation similarly slow responding and increase error rate. Inhibition of the STN during surprise also increased error rate, but blocked the surprise-effect on response speed. These results support a more general role for the STN in stopping not only action but also cognition.

## Introduction

The subthalamic nucleus (STN) is a substriatal nucleus of the basal ganglia that gets input from the striatum via the indirect pathway and from the cortex via the hyperdirect pathway. The STN is composed principally of glutamatergic neurons that densely project to the inhibitory output structures of the basal ganglia: the internal globus pallidus (GPi; in rodents called entopeduncular nucleus) and substantia nigra pars reticulata (SNr). Because STN activity is thought to increase inhibition of downstream targets in the motor thalamus and superior colliculus, classical models of basal ganglia function have posited that activity in the STN is anti-kinetic, serving as a brake on behavioral output (Mink, 1996).

A wealth of evidence implicates the STN in rapidly suppressing behavior in response to external signals (Jahanshahi et al., 2015; Wessel et al., 2016; Schmidt and Berke, 2017; Li et al., 2020). For example, the STN is activated by signals to stop an initiated response as shown by human fMRI (Aron and Poldrack, 2006), local field potential recording (Ray et al., 2012; Wessel et al., 2016), and single-unit recordings in humans (Bastin et al., 2014; Benis et al., 2016), non-human primates (Isoda and Hikosaka, 2008; Pasquereau and Turner, 2017), and rodents (Schmidt et al., 2013). Moreover, we recently showed that optogenetic activation of the STN is sufficient to rapidly suppress natural licking behavior, and that STN inhibition can blunt the interruptive effects of surprise on the same behavior (Fife et al., 2017).

In addition to its role in suppressing movement, several lines of evidence suggest a role for the STN in interrupting cognitive processes. In a series of experiments, Aron and colleagues found that surprising events impair accuracy in a working memory assay, slow reaction speed, increase γ and β oscillations in STN, and activate the same frontal cortical neural signatures as does action-stopping (Wessel and Aron, 2017). More recently they also showed that volitional suppression of thoughts (i.e., preventing retrieval of an unwanted long-term memory) also recruits a similar frontal cortical signature as does action-stopping (Castiglione et al., 2019). Second, lesioning or pharmacological inhibition of rat STN leads to increased errors and compulsive responding in an attention task (Baunez and Robbins, 1997, 1999). Third, STN deep brain stimulation (DBS) has cognitive effects in Parkinson’s disease patients (Parsons et al., 2006; Sáez-Zea et al., 2012; Gourisankar et al., 2018). Lastly, STN activity has been linked to the prevention of memory encoding (Zavala et al., 2017).

Based on such data we posit a model wherein surprising stimuli trigger a burst in STN activity that not only slows or halts an ongoing action, but also interrupts cognitive processes. To test this hypothesis, we developed a delayed-match-to-position (DMTP) assay that required mice to remember and then respond at a visuospatial location over a delay period and was interruptible by a surprising sensory stimulus. We then used optogenetic excitation or inhibition to control STN activity during the delay and test whether manipulations in STN activity could induce or prevent interruptions in DMTP performance.

## Materials and Methods

### Animals

Homozygous *Slc17a6^IRESCre^* (vesicular glutamate transporter 2; VGLUT2-Cre) mice (Vong et al., 2011) were obtained (The Jackson Laboratory, #016963), maintained in-house on a C57Bl/6 background, and used in accordance with guidelines established by the Institutional Animal Care and Use Committee. Mice were maintained on a 12/12 h light/dark cycle in a temperature-controlled and humidity-controlled environment, group-housed by sex in plastic cages (maximum five mice/cage) with lofts and cotton nestlets for enrichment. Food and water were available *ad libitum*, except where noted. Both male and female mice (more than six weeks) were used in approximately equal proportion. Behavioral testing and training began 1–3 h after lights on and continued until the entire cohort had been run. Mice were trained/tested using four operant chambers and the cages were cleaned between testing sessions. Male and female mice were run concurrently using separate boxes. Each figure represents an independent cohort of mice, except Figure 5*C*,*D*, which represent data collected from subjects first used in Figure 3.

Anesthetized mice were infused with 400nl of recombinant adeno-associated virus (AAV) and optic fibers implanted into STN as described (Fife et al., 2017). Briefly, Cre-dependent expression of yellow fluorescent protein (YFP)-tagged Channelrhodopsin-2 (ChR2; H134R), YFP-tagged halorhodopsin (eNpHR 3.0), or YFP (controls) was achieved with rAAV5-EF1α-DIO-ChR2:YFP (7 × 10^12 genomes/ml), rAAV5-EF1α-DIO-eNpHR3.0:YFP (4 × 10^12), or AAV5-EF1α-DIO-EYFP (6.5 × 10^12); all AAVs were obtained from University of North Carolina Vector core. Mice were allowed to recover for more than two weeks before initiating training, and testing was conducted more than eight weeks after surgery. Following behavioral experiments histology was performed as described (Fife et al., 2017). Sections from each animal were examined for native fluorescence and implant site in STN. One mouse (ChR2; Fig. 3) was excluded because of spread of virus throughout thalamus; one mouse (Halo; Fig. 4) was excluded because of optic fiber misplacement caudal to STN; one mouse (eYFP; Fig. 4) was excluded because of genotype error.

### DMTP

Group-housed mice were food restricted to ∼85% body weight throughout training and testing. Operant chambers (Med Associates, ENV-352-2W) were arranged with three nosepoke holes on the left wall. The right wall held a liquid reward port connected to a pump that delivered 10% sucrose, a single LED strip for low-level illumination throughout experiment, a clicker to signal reward delivery, one speaker to deliver standard cue (250-ms 3.3-kHz tone at 80 db) and another to deliver the novel cue (500-ms white noise at 80 db). An overhead house light was also illuminated during the novel cue.

Training and testing sessions were 45 min and conducted during the light cycle. Each trial began with a single nosepoke-hole illuminated. The mouse was first tasked with sampling this hole causing the illumination to be extinguished for a variable delay. During training 1-, 2-, 4-, 6-, and 10-s delays were selected at equal probability on a variable schedule. In each case the standard cue was played 500 ms before the end of the delay period. At the end of the delay all three nosepoke holes were illuminated and the mouse could earn a reward by re-poking the sampled hole within an 8-s response window. Correct responding led to 25 μl of sucrose reward that was signaled by a solenoid clicker and illumination of the reward port. Reward retrieval extinguished the illuminated reward port and immediately led to the initiation of the next trial. Errors included selection of the incorrect nosepoke-hole or omission of a response within the 8-s response window, both of which led to a 10-s timeout in which all illumination was extinguished, followed by initiation of the next trial. When responses were made, latency was measured from onset of response period (when all three nosepoke-holes were illuminated) to the nosepoke response.

**Figure 1.**
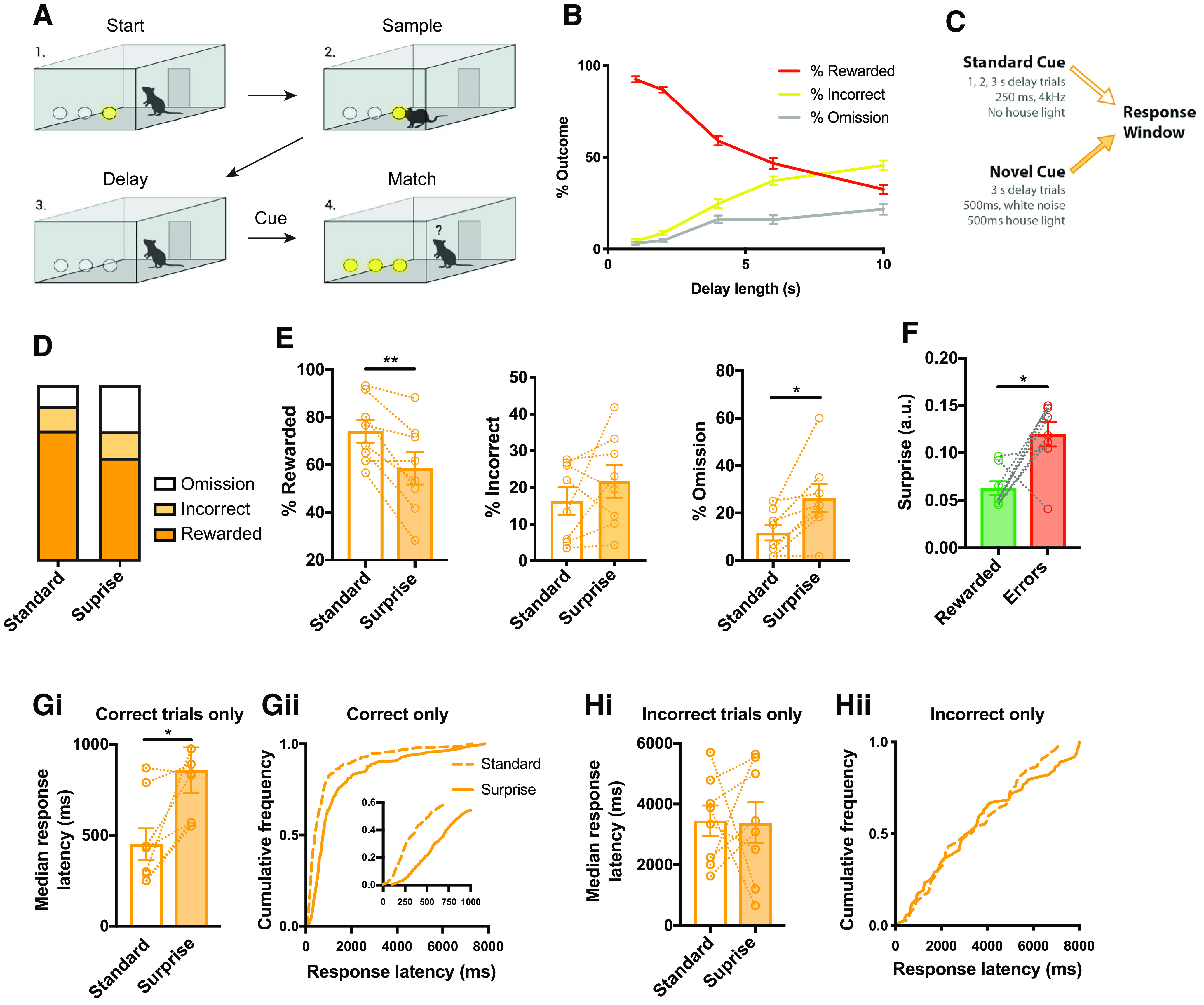
Surprise impaired accurate re-selection of the target nose hole and slowed responding on a delayed match to position task. ***A***, Schematic of task. ***B***, The trial outcomes as a function of delay shows decreasing reward rate and increasing error rate with longer delay. ***C***, On the surprise version of task, the standard cue was replaced with a novel cue 50% of all 3-s trials. ***D***, Surprise altered the pooled distribution of trial outcomes. ***E***, The reward was reduced by surprise, the rate of incorrect responses was not significantly changed, and the omission rate was increased. ***F***, Bayesian surprise values of novel trials split by outcome show that more surprising trials led to more response errors. ***G_i_***, Surprise increased the mean median response latency on correct trials and (***G_ii_***) led to a rightward shift of pooled latencies. ***H_i_***, Surprise did not change mean median latency on trials were an incorrect response was made, (***H_ii_***) nor did it lead to a change in the pooled distribution of response latencies; *p < 0.05, **p < 0.01 by two-tailed paired *t* test.

**Figure 2.**
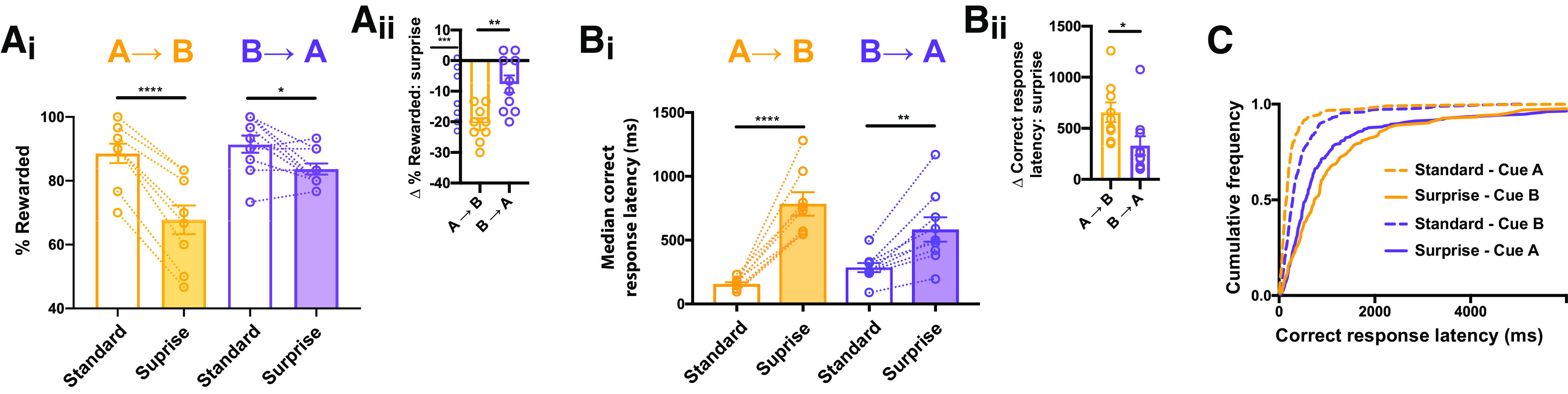
Surprise decrements performance because the novel cue has higher salience and violates an expectation. ***A_i_***, In both the A→B and B→A versions of the task, the novel cue elicits a significant decrease in the reward rate, (***A_ii_***) but the effect was larger when the novel cue was more salient (A→B). ***B_i_***, In both versions of the task, the novel cue elicits a significant increase in the latency to make a correct response, (***B_ii_***) but the effect was larger when the novel cue was more salient. ***C***, Surprise induced a rightward shift in the distribution of pooled correct response latencies in both versions of the task; *p < 0.05, **p < 0.01, ****p < 0.0001 by Holm–Sidak *post hoc* test (***A_i_***, ***B_i_***) or two-tailed paired *t* test (***A_ii_***, ***B_ii_***).

Mice were trained until the mean % rewarded of all mice was >50% and there was some cohort-to-cohort variability in the number of sessions required to meet this (means ranged between 31 and 36 sessions between cohorts). Mice were trained 5 d/week and tested on consecutive days until completing the requisite trial number for each experiment. Procedures for testing effects of novel cue (surprise; Fig. 1) were similar, but only used delays of 1, 2, and 3 s (each with standard cue) as well as trials with 3-s delays using the novel cue (also presented 500 ms before end of the delay period). Each of these four trial types occurred at equal probability on a variable schedule so that each trial type (1 s standard, 2 s standard, 3 s standard, 3 s novel) occurred at 25% probability. Data shown represent 3-s trial types only. For both Figures 1, 2, sessions were repeated on consecutive days (3–7 d) until we collected at least 30 trials of each type for each mouse.

**Figure 3.**
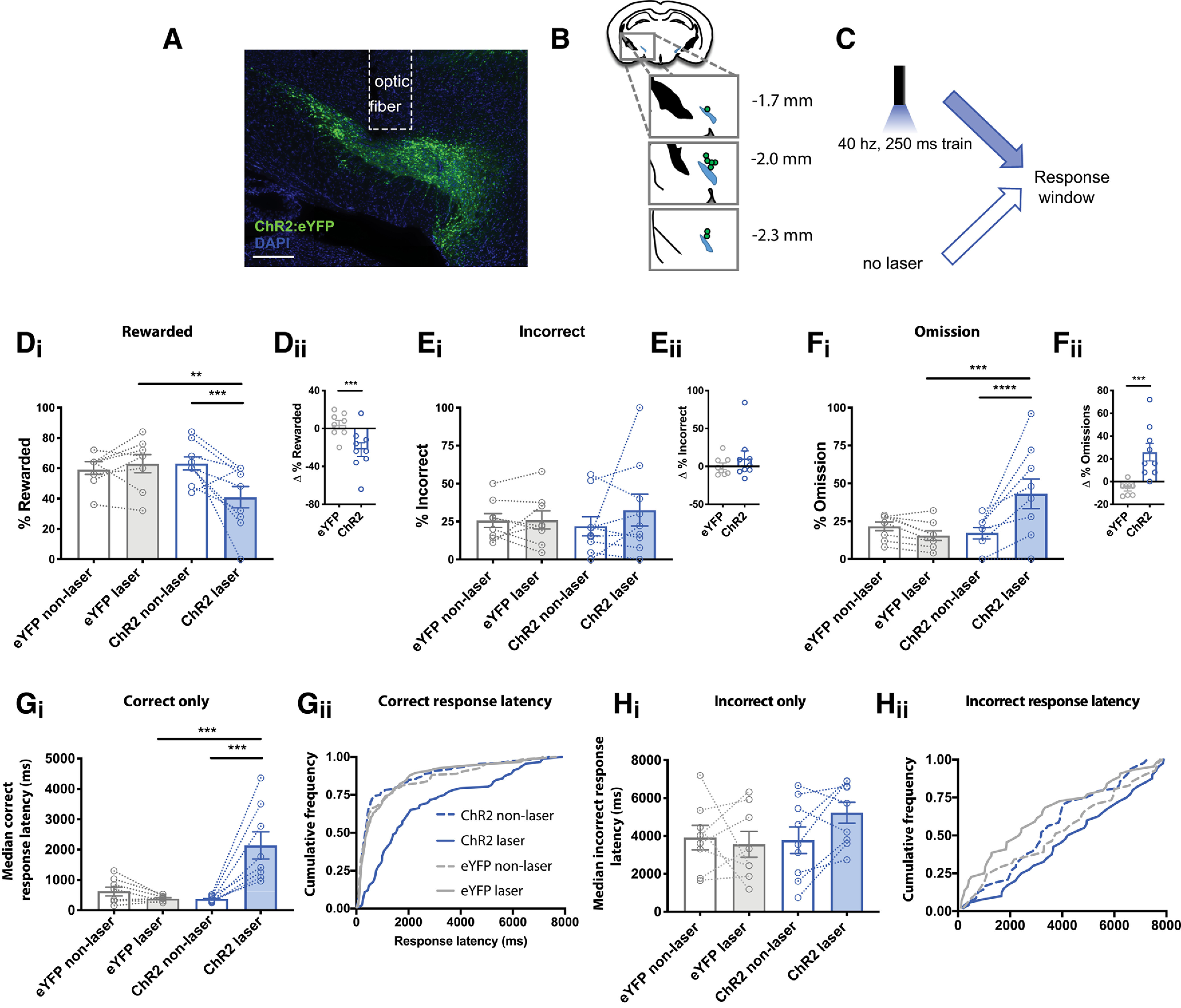
Optogenetic stimulation of the STN impaired accurate re-selection of the target nose hole and slowed responding. ***A***, Exemplar showing unilateral expression of ChR2:eYFP in STN and fiber optic implant placement. Scale bar: 200 μm. ***B***, Map illustrating location of fiber optic implant placements. ***C***, Schematic of experimental design comparing laser to non-laser trials. ***D_i_***, ***D_ii_***, In ChR2-expressing mice, but not eYFP-expressing controls, laser decreased reward rate. ***E_i_***, ***E_ii_***, The incorrect response rate was not significantly changed by laser stimulation. ***F_i_***, ***F_ii_*,** The omission error rate was increased by laser in the ChR2-expressing, but not eYFP control, mice. ***G_i_***, In ChR2-expressing mice, photostimulation increased the mean median and (***G_ii_***) induced a rightward shift in the pooled distribution of correct response latencies. ***H_i_***, ***H_ii_***, Photostimulation did not alter the latency to make an incorrect response; *p < 0.05, **p < 0.01, ***p < 0.001, ****p < 0.0001 by Holm–Sidak *post hoc* test (***D_i_***, ***F_i_***, ***G_i_***) or two-tailed paired *t* test (***D_ii_***, ***F_ii_***).

Procedures were similar for testing the effects of optogenetic stimulation (ChR2; Fig. 3), except the auditory cue signaling the initiation of the sample phase was excluded throughout both training and test. During testing the standard and novel cues were replaced by non-laser and laser trials, respectively. In the final training session before test, mice were tethered to the laser via implanted fiber/ferrule and patch cables were coupled to an optical commutator (Doric) to allow them to acclimate without any laser delivery. On laser trials the computer triggered a DPSS laser (473 nm, Shanghai or OEM Laser) to deliver photostimulation (250-ms train of 10-ms pulses at 40 Hz at 10 mW). Modeling clay was applied at the junction of the patch cable to block all light. Sessions were repeated on consecutive days (2–6 d) until we collected at least 25 trials of both laser and non-laser trial for each mouse.

**Figure 4.**
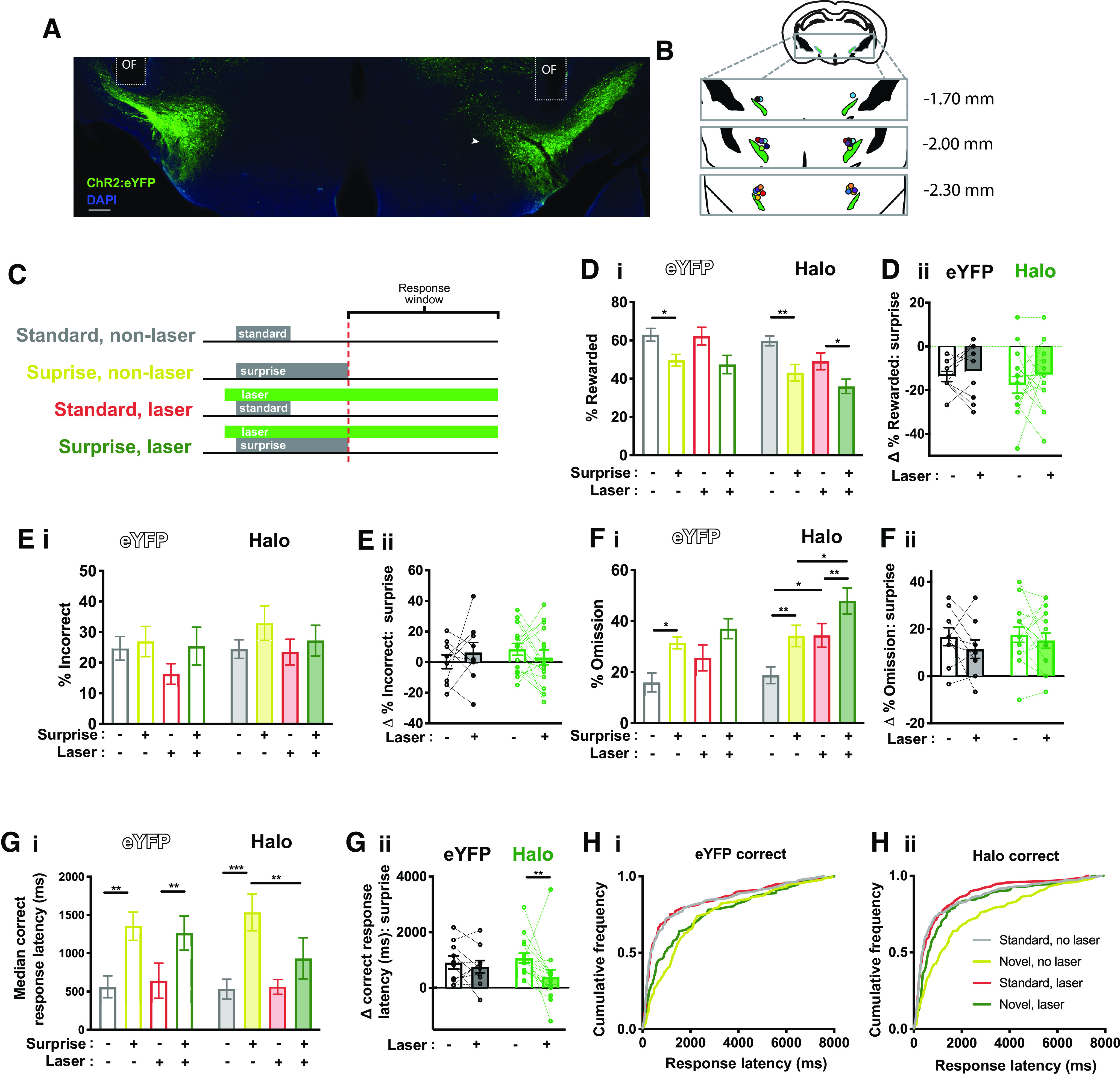
Optogenetic inhibition of the STN blunted surprise-induced slowing. ***A***, Exemplar showing bilateral expression of Halo:eYFP in the STN. ***B***, Map of fiber optic implant placements. ***C***, Schematic of experimental design. ***D_i_***, Both eYFP-expressing and Halo-expressing mice performed worse on surprise trials and (***D_ii_***) the effect of surprise was unchanged by optogenetic inhibition. ***E_i_***, ***E_ii_***, Optogenetic inhibition did not influence the effect of surprise on incorrect response rate. ***F_i_***, Both eYFP-expressing and Halo-expressing mice increased omission rate on surprise trials, (***F_i_***) and the effect of surprise was unchanged by optogenetic inhibition ***G_i_***, ***G_ii_***, Both eYFP-expressing and Halo-expressing mice were slower to make a correct response on surprise trials, but this effect was greatly diminished by optogenetic inhibition. ***H_i_***, eYFP-expressing mice showed a rightward shift in the pooled distribution of correct response latencies following the novel cue in both laser-on and laser-off conditions, but (***H_ii_***) Halo-expressing mice showed this rightward shift only in the laser-off condition; *p < 0.05, **p < 0.01, ***p < 0.001 by Holm–Sidak *post hoc* test.

Procedures for testing the effects of optogenetic inhibition (Fig. 4) were similar to those used for testing the effects of standard versus novel cue. Except that, during testing, green laser light was delivered on 50% of the 3-s trials. Thus, the following 3-s trials each occurred at an overall probability of 12.5%: standard cue without laser, novel cue without laser, standard cue with laser, novel cue with laser. As with previous experiments, 50% of the trials were 1- or 2-s trials, but we present only data comparing the 3-s trials. In their final training session before test, mice were tethered to the laser to acclimate to the patch cable. On laser trials the computer triggered a DPSS laser (532 nm, Shanghai Laser) to deliver green light; 10-mW illumination delivered 50 ms before the standard or surprise cues and continuing until the end of the trial. Thus, the laser on times ranged from 0.55 to 8.55 s. Sessions were repeated on consecutive days (4–13 d) until we collected at least 30 trials of each trial type for each mouse.

It was noted that mice in both optogenetic experiments (Figs. 3, 4) performed at somewhat lower reward rates than those observed for mice without optogenetic manipulation (Figs. 1, 2). This is likely because of the tethering required for light delivery, as their performance was indistinguishable in the final training sessions before tethering.

### Open field assay

Mice were tethered to lasers as described above, placed in an open-field arena (50 × 50 cm) and received the first delivery of laser light after 45 s. For, the ChR2 experiments mice received a brief 250-ms train at 40 Hz of 10-mW blue light every 45 s for 20 cycles. In the Halo version of this experiment, a 15-s continuous beam of 10-mW green light was delivered every 45 s for 40 cycles. The sessions were repeated three times each, once every four weeks, and data were averaged across the sessions.

### Statistics

Data are presented as individual points and means ± SEM, except where noted, and subjected to statistical procedures stated. Response latency data were not normally distributed and log transformed for statistical testing. Statistical significance was set at *p* < 0.05 and Prism GraphPad or SPSS was used.

## Results

### Surprise increased errors and slowed responding on matching task

To causally test how STN activity could impact cognitive performance we developed a DMTP task that requires mice to sustain attention and motivation to solve a variable visuospatial problem (Fig. 1*A*). Each trial began with the illumination of one of three nosepoke holes. The mouse then poked the illuminated hole, which caused the light to extinguish for a variable delay. A cue then indicated the end of the delay period, and 500 ms later all three holes were illuminated. Mice earned a reward for correctly re-poking the sampled hole within 8 s. Poking the incorrect hole or failing to respond within 8 s (omission) were considered errors and resulted in a 10-s timeout.

After training, mice were tested with a variable delay of 1–10 s, with longer delays resulting in a reduced reward rate (Fig. 1*B*). The erosion of performance across delay was manifest by an increase in both incorrect responses and response omissions, consistent with DMTP performance providing a cognitive measure of sustained goal maintenance. Throughout the remaining experiments all manipulations were done on 3-s trials to yield an intermediate level of performance from which both impairments and enhancements could be detected.

To test the disruptive effect of surprising events, we replaced the standard cue with a novel (surprising) cue on 50% of the 3-s delay trials, which themselves represented 50% of the total trials (see Materials and Methods; Fig. 1*C*). Replacement of the standard cue with the novel cue shifted the distribution of trial outcomes, reduced the proportion of correct trials and increased the proportion of incorrect and omission errors (Fig. 1*D*), resulting in a reduction in the fraction of trials that were rewarded (*n* = 8, two-tailed paired *t* test, *p* = 0.006; Fig. 1*E*). There was not a significant increase in the percentage of incorrect responses (*p* = 0.26), thus the reduced reward rate was mainly accounted for by an increase in omission errors (*p* = 0.012; Fig. 1*E*).

Surprising events (novel cues) slow reaction times in human subjects (Wessel and Aron, 2013), and we tested for a similar effect here. Because correct responses were much more rapid than incorrect responses, even in the standard condition, correct and incorrect responses were considered separately. We found that the mean median latency to make a correct response was increased significantly by surprise (*n* = 8, two-tailed paired *t* test, *p* = 0.024; Fig. 1*Gi*). When pooled across mice, the distribution of response latencies showed a rightward shift following surprise [Kolmogorov–Smirnov (KS) test, *p* < 0.0001; Fig. 1*Gii*]. Importantly, this rightward shift was present at the shortest latencies (Fig. 1*Gii*, inset), indicating that even the most rapid responses were slower following surprise; thus, the slower response time was not merely the result of an increase in the number of very long latency responses that could correspond to “guessing.” The response speed on incorrect trials was unaffected by surprise as measured by mean median response latency (*p* = 0.70; Fig. 1*Hi*) or pooled response latencies (*p* = 0.56; Fig. 1*Hii*). Together, these results show that following a surprising event, mice were less likely to respond, but that when they did respond accurately their responses were slower.

### Sensory salience and violation of expectation both contributed to the effect of surprise

Relative to the standard cue (referred to here as cue A), the novel cue (cue B) was not only unexpected, but also longer (500 vs 250 ms), broadband (white noise vs tone), and paired with a visual component (house light). To determine whether the effect on DMTP performance was driven only by the heightened sensory salience of the novel cue, or whether violation of expectation could also drive the effect, we trained two new cohorts. In the first cohort, mice were trained with cue A and then on test day probed with cue B (A→B, as before), while in the second cohort of mice these cues were swapped (B→A).

ANOVA was run with one within-subject factor (surprise) and one between-subjects factor (cohort). As before, we found an effect of surprise, but also an effect of cohort, and an interaction between surprise and cohort (surprise, *F*_(1,17)_ = 66.7, *p* < 0.0001; cohort, *F*_(1,17)_ = 5.3, *p* = 0.034; surprise × cohort, *F*_(1,17)_ = 14.1, *p* = 0.002; Fig. 2*Ai*). The interaction indicates a stronger impact of surprise on cohort A→B compared with cohort B→A; further supported by an analysis showing a significantly larger reduction in reward rate in A→B (*t* test, *p* = 0.002; Fig. 2*Aii*).

We also compared the effects of surprise on the latency to make a correct response, and again found a main effect of surprise, and this effect was observed in both cohort A→B and B→A (ANOVA; surprise, *F*_(1,17)_ = 246, *p* < 0.0001; cohort, *F*_(1,17)_ = 0.11, *p* = 0.74; surprise × cohort *F*_(1,17)_ = 35.4, *p* < 0.0001; Fig. 2*Bi*). However, the significant interaction between surprise and cohort indicates a bigger effect of surprise in cohort A→B compared with cohort B→A, and indeed surprise-induced slowing was larger in magnitude in group A→B (*p* = 0.03; Fig. 2*Bii*). The distribution of pooled response latencies also revealed an effect of surprise in both A→B (KS, *p* < 0.0001; Fig. 2*C*) and B→A (*p* < 0.0001), but the KS D’ in A→B (0.60) was nearly twice that of B→A (0.32). Thus, for both reward rate and response speed, the impact of surprise cue was greater when the sensory salience of the novel cue was higher (i.e., A→B).

Critically, however, even a novel cue with reduced sensory salience (i.e., B→A) increased error rate and slowed responding. This shows that violation of expectation was sufficient to perturb performance in this assay.

### Optogenetic stimulation of STN increased omission errors and slowed responding

Our hypothesis is that surprising events are ethologically relevant stop signals that recruit STN to slow, pause, or interrupt behavior and cognition. If correct, then directly activating the STN should mimic the effect of surprise in the DMTP assay. To test this, we used an optogenetic approach to stimulate STN, similar to the approach prior employed to interrupt licking behavior (Fife et al., 2017). A Cre-dependent AAV vector was unilaterally injected into the STN of VGLUT2-Cre mice to selectively express either Channelrhodopsin-2 fused to eYFP (ChR2; *n* = 9) or eYFP control (*n* = 8) in the STN (Fig. 3*A*); and optic fibers were implanted to deliver light just dorsal to the site of injection (Fig. 3*B*).

The task was as in Figure 1, but standard and novel cues were replaced with non-laser and laser trials, respectively (Fig. 3*C*). Laser trials consisted of 10 pulses at 40 Hz delivered 500 ms before the expiration of the delay period, the same timing as cue/surprise delivery in Figure 1.

Similar to surprise, optogenetic stimulation of STN caused a marked decrease in the reward rate of ChR2-expressing mice compared with non-laser trials (within subject), or compared with laser trials in eYFP-expressing control mice. We found an interaction between laser and virus indicating that ChR2-expressing mice are selectively effected by laser delivery (ANOVA; laser, *F*_(1,15)_ = 4.2, *p* = 0.06; virus, *F*_(1,15)_ = 1.9, *p* = 0.18; laser × virus, *F*_(1,15)_ = 8.7, *p* = 0.01; Fig. 3*Di*). Moreover, the change in reward rate induced by laser was significantly greater in the ChR2 compared with control mice (two-tailed unpaired *t* test, *p* = 0.006; Fig. 3*Dii*). We detected no change in the proportion of incorrect responses with optogenetic activation of STN (virus, *F*_(1,15)_ = 0.02, *p* = 0.88; laser *F*_(1,15)_ = 0.94, *p* = 0.35; laser × virus interaction, *F*_(1,15)_ = 0.82, *p* = 0.38; Fig. 3*Ei*) and the effect of laser did not differ between virus groups when compared directly (*p* = 0.37; Fig. 3*Eii*). Instead, optogenetic activation led to an increase in the rate of omission errors (laser *F*_(1,15)_ = 5.5, *p* = 0.03; virus, *F*_(1,15)_ = 2.6, *p* = 0.13; laser × virus interaction, *F*_(1,15)_ = 14.8, *p* = 0.002; Fig. 3*Fi*). This can also be observed as an increase in omission errors induced by laser in the ChR2 group (*p* = 0.002; Fig. 3*Fii*).

Similar to the effect of surprise, optogenetic activation of the STN also led to response slowing on correct trials (ANOVA; virus, *F*_(1,15)_ = 9.8, *p* = 0.007; laser *F*_(1,14)_ = 25.7, *p* = 0.0002; laser × virus interaction *F*_(1,15)_ = 55.3, *p* < 0.0001; Fig. 3*Gi*). This was also detected as a rightward shift in the pooled distribution of response latencies (KS test, *p* < 0.0001; Fig. 3*Gii*). The effect was apparent even at short latencies, indicating that, similar to the effect of surprise, the slowing effect was not simply because of an increased proportion of long-latency responses. The response latency on incorrect trials was unaffected by optogenetic stimulation of STN [ANOVA; virus, *F*_(1,15)_ = 0.47, *p* = 0.50; laser, *F*_(1,13)_ = 0.76, *p* = 0.40; laser × virus interaction *F*_(1,13)_ = 2.8, *p* = 0.12 (Fig. 3*Hi*); *p* = 0.11 (Fig. 3*Hii*)]. Thus, both surprise and optogenetic activation of STN reliably increased the rate of omission errors and resulted in slower responding when correct responses were made.

### Optogenetic inhibition of the STN prevented the slowing in response to surprise

Our hypothesis is that surprise can slow or interrupt behavior and cognition, in part, by recruiting STN. If correct, then inhibiting the STN should reduce the effects of surprise. To test this, we bilaterally expressed Halorhodopsin:eYFP (Halo; *n* = 15; Fig. 4*A*) or eYFP controls (*n* = 9) in the STN of VGLUT2-Cre mice, and bilaterally implanted optic fibers just dorsal (Fig. 4*B*).

As in Figure 1, the standard-cue was replaced with the novel-cue (i.e., surprise) in 50% of the 3-s-delay trials and 25% of trials were surprise trials. Here, however, for 50% of each 3-s trial type the laser was activated 50 ms before cue presentation, and the laser remained on until the end of the trial (Fig. 4*C*).

ANOVA was first performed with two within-subject factors (surprise, laser) and one between-subjects factor (virus). As before, surprise reduced the reward rate, there was also a main effect of laser, but no other effects nor interactions were detected (surprise, *F*_(1,22)_ = 45.5, *p* < 0.0001; laser, *F*_(1,22)_ = 4.4, *p* = 0.048; virus, *F*_(1,22)_ = 3.8, *p* = 0.06; laser × virus *F*_(1,22)_ = 2.2, *p* = 0.15; laser × surprise *F*_(1,22)_ = 0.06, *p* = 0.81; surprise × virus *F*_(1,22)_ = 0.04, *p* = 0.85; laser × virus × surprise *F*_(1,22)_ = 0.36 *p* = 0.55; Fig. 4*Di*). Because of the effect of laser on reward rate, we conducted a two-way ANOVA on just the Halo mice and found an overall effect of surprise and laser, but no interaction between the two factors (surprise, (*F*_(1,14)_ = 30.2, *p* < 0.0001; laser, *F*_(1,14)_ = 7.1, *p* = 0.02; surprise × laser. *F*_(1,14)_ = 0.6, *p* = 0.46). The lack of interactions suggest that the effect of laser was independent of surprise. Indeed, when we isolated the effect on reward rate induced by surprise, we found it was not changed by optogenetic inhibition (two-way-ANOVA; virus, *F*_(1,22)_ = 0.33, *p* = 0.57; laser, *F*_(1,22)_ = 1.1, *p* = 0.30; virus × laser, *F*_(1,22)_ = 0.20, *p* = 0.66; Fig. 4*Dii*). Thus, contrary to our hypothesis, STN inhibition did not blunt the effects of surprise on trial outcome (reward rate or error rate), rather inhibiting STN led to a modest reduction in reward rate independent of trial type.

We also detected an overall effect of surprise on % incorrect as well as an overall effect of laser but no interactions (surprise, *F*_(1,22)_ = 7.1, *p* = 0.014; laser, *F*_(1,22)_ = 4.8, *p* = 0.039; virus, *F*_(1,22)_ = 0.4 *p* = 0.54; laser × virus *F*_(1,22)_ = 0.18, *p* = 0.6; laser × surprise *F*_(1,22)_ = 0.1, *p* = 0.81; surprise × virus *F*_(1,22)_ = 0.01, *p* = 0.92; laser × virus × surprise *F*_(1,22)_ = 0.2 *p* = 0.21; Fig. 4*Ei*). When we isolated the effect on incorrect rate induced by surprise, we found it was not changed by optogenetic inhibition (two-way-ANOVA; virus, *F*_(1,22)_ = 0.2, *p* = 0.66; laser, *F*_(1,22)_ = 0.0, *p* = 0.95; virus × laser, *F*_(1,22)_ = 1.4, *p* = 0.25; Fig. 4*Eii*). As with reward rate and incorrect rate, there were main effects of surprise and laser on omission rate, but no interactions (surprise, *F*_(1,22)_ = 7.1, *p* = 0.014; laser, *F*_(1,22)_ = 4.8, *p* = 0.039; virus, *F*_(1,22)_ = 0.4 *p* = 0.54; laser × virus *F*_(1,22)_ = 0.2, *p* = 0.6; laser × surprise *F*_(1,22)_ = 0.1, *p* = 0.81; surprise × virus *F*_(1,22)_ = 0.0, *p* = 0.92; laser × virus × surprise *F*_(1,22)_ = 0.2, *p* = 0.21; Fig. 4*Fi*). Again, when we isolated the effect on omission rate induced by surprise, we found it was not changed by optogenetic inhibition (two-way-ANOVA; virus, *F*_(1,22)_ = 1.6 *p* = 0.22; laser, *F*_(1,22)_ = 2.0, *p* = 0.17; virus × laser, *F*_(1,22)_ = 0.3, *p* = 0.62; Fig. 4*Fii*). These results are consistent with our STN inhibition being ineffective in blunting the effect of surprise on trial outcome.

In contrast to the effects on trial outcome (reward, incorrect, and omission rate), our analyses of response latency showed that STN inhibition did blunt the effect of surprise in a manner consistent with our hypothesis (Fig. 4*Gi*). We found a main effect of surprise, an interaction between laser and surprise and, importantly, a three-way interaction between surprise, laser, and virus—but no other significant effects or interactions were detected (ANOVA; surprise, *F*_(1,22)_ = 44.4, *p* < 0.0001; laser, *F*_(1,22)_ = 1.4, *p* = 0.24; virus, *F*_(1,22)_ = 0.2, *p* = 0.69; surprise × laser, *F*_(1,22)_ = 8.8, *p* = 0.007; laser × virus *F*_(1,22)_ = 0.8, *p* = 0.57; surprise × virus *F*_(1,22)_ = 0.3, *p* = 0.57; laser × virus × surprise *F*_(1,21)_ = 4.9, *p* = 0.039).

Separate two-way ANOVAs revealed that the three-way interaction was driven by an interaction between surprise and laser in the Halo group (ANOVA; surprise, *F*_(1,14)_ = 27.7, *p* = 0.0001; laser, *F*_(1,14)_ = 2.6, *p* = 0.13; surprise × laser *F*_(1,14)_ = 16.7, *p* = 0.001) that was absent in the eYFP group (ANOVA; surprise, *F*_(1,8)_ = 16.4, *p* = 0.004; laser, *F*_(1,8)_ = 0.0, *p* = 0.90; surprise × laser *F*_(1,8)_ = 0.0, *p* = 0.87). While surprise reliably slowed responding on correct trials in the eYFP animals and in the Halo animals in the absence of laser, laser significantly blunted the increase in latency for the Halo group (ANOVA; laser, *F*_(1,22)_ = 8.8, *p* = 0.007; virus, *F*_(1,22)_ = 0.3, *p* = 0.58; laser × virus, *F*_(1,22)_ = 5.5 *p* = 0.03; Fig. 4*Gii*).

The slowing effect of surprise was reduced by laser in 87% of Halo mice, but only 56% (approximately chance) of eYFP mice. We also observed a leftward shift of laser on the correct response latency distribution on surprise trials in Halo mice (KS, *p* = 0.0003; Fig. 4*Hi*), and this effect was absent in eYFP mice (*p* = 0.12; Fig. 4*Hii*). In sum, photoinhibition of the STN did not mitigate the disruptive effect of surprise on reward rate or error rate but led to a modest reduction in reward rate independent of surprise, and led to a significant reduction in the slowing effect of surprise on response time.

### STN excitation and inhibition in the open field

**Figure 5.**
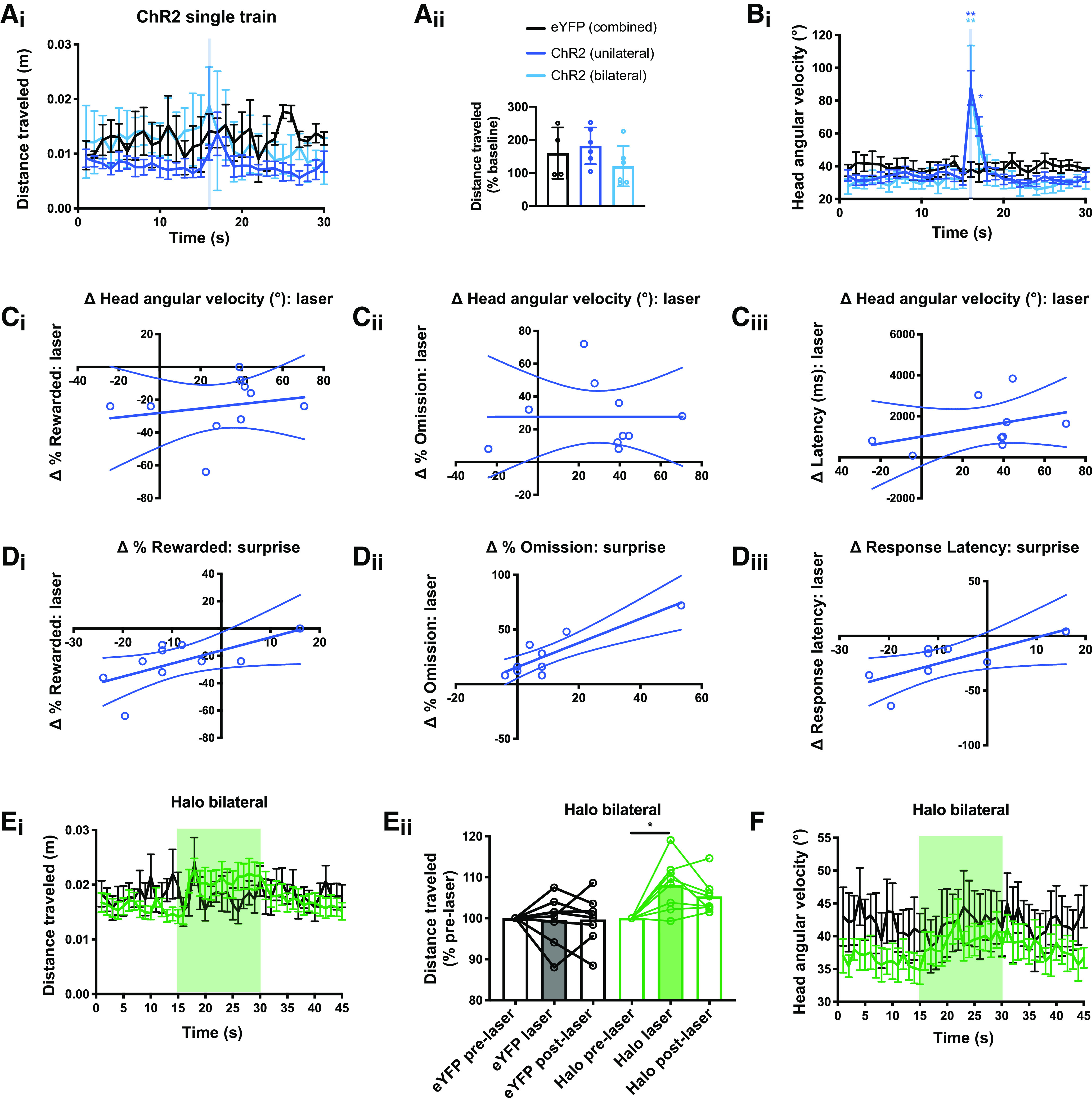
Optogenetic activation and inhibition of the STN in an open-field assay. ***A_i_***, Time course of distance traveled in the open field with unilateral or bilateral optogenetic activation of the STN (10-pulse train of blue light, 40 Hz, onset at *t* = 15 s). ***A_ii_***, No difference in distance traveled was detected between groups. ***B***, Optogenetic activation did induce a transient change in head angular velocity of ChR2-expressing mice. ***C***, Mice from Figure 3 were subsequently tested in the open-field assay. There was no correlation between the magnitude of the head angular velocity change induced by optogenetic stimulation and the change in (***C_i_***) reward rate, (***C_ii_***) omission rate, or (***C_iii_***) correct response latency. ***D***, Mice from Figure 3 were subsequently tested in the DMTP with surprise assay. There was a significant correlation between the (***D_i_***) change in reward rate induced by optogenetic activation and surprise, (***D_ii_***) as well as for the change in omission rate, but (***D_iii_***) not for the change in correct response latency. ***E_i_***, Time course of distance traveled in the open field with bilateral optogenetic inhibition of the STN (15 s of continuous green light, onset at *t* = 15). ***E_ii_***, Optogenetic inhibition produced a small but significant increase in distance traveled during the laser period compared with the pre-laser period. ***F***, Time course of head angular velocity in the open field showed no apparent effect induced by optogenetic inhibition of the STN; *p < 0.05 by Holm–Sidak *post hoc* test.

We next tested how the same pattern of optogenetic stimulation (ChR2) or inhibition (Halo) influenced open-field activity in new cohorts of mice. We first tested the effects of both unilateral and bilateral optogenetic stimulation via ChR2 and used stimulus conditions that matched those used in the DMTP assay (10 pulses delivered at 40 Hz; Fig. 5*Ai*). To focus on changes in distance traveled around the time of laser delivery, we normalized the 4-s post-stimulus activity to the 4-s pre-stimulus activity within animal and found no effect of optogenetic stimulation (one-way ANOVA; *F*_(2,13)_ = 1.4, *p* = 0.27; Fig. 5*Aii*). However, brief STN activation did provoke a transient change in angular velocity of the head (two-way ANOVA; time, *F*_(3.4,43.7)_ = 21.7, *p* < 0.0001; virus, *F*_(2,13)_ = 1.4, *p* = 0.29; time × virus, *F*_(58,377)_ = 6.0, *p* < 0.0001; Fig. 5*B*). This increase in head angular-velocity may reflect a brief involuntary movement that appeared to principally involve a change in head orientation, always in the ipsiversive direction when stimulation was delivered unilaterally. However, the behavior was present, and similar in magnitude, whether stimulation was delivered unilaterally or bilaterally, and appeared generally consistent within animal, but varied between animals.

To test whether the change in angular velocity may relate to the effects observed in the DMTP assay, the ChR2-expressing mice from Figure 3 were subsequently tested in the open-field assay and we ran correlational analyses between measures derived from each test. We found no significant within-subject correlation between the angular velocity change detected in open field and the effect of laser on reward rate (Pearson, *R*^2^ = 0.02, *p* = 0.69; Fig. 5*Ci*), omission error rate (*R*^2^ = 0.03, *p* = 0.66; Fig. 5*Cii*), or correct response latency (*R*^2^ = 0.29, *p* = 0.14; Fig. 5*Ciii*) in the DMTP. Where any tendency was observed, mice with smaller changes in angular velocity showed greater changes in DMTP. These data suggest that changes in angular velocity were not driving behavioral effects of optogenetic stimulation in the DMTP.

We next tested these same ChR2-expressing mice from Figure 3 in the unexpected event assay similar to that used in Figure 2 for correlational analyses of ChR2-induced and surprise-induced effects on DMTP. Indeed, we found significant within-subject correlations between the optogenetic and surprise-induced effects on reward rate (Pearson, *R*^2^ = 0.52, *p* = 0.044; Fig. 5*Di*) and omission error rate (*R*^2^ = 0.77, *p* = 0.002; Fig. 5*Dii*), but not on response latency (*R*^2^ = 0.18, *p* = 0.70; Fig. 5*Diii*). Thus, the mice that were most interruptible by surprise also tended to be most interruptible by STN activation.

Finally, we tested the effects of STN inhibition. Since the duration of optogenetic inhibition in the DMTP was variable (i.e., dependent on trial by trial response latency) we delivered 15-s continuous light (Fig. 5*Ei*). We found that sustained bilateral inhibition of the STN produced a small but significant increase in distance traveled (two-way ANOVA; virus, *F*_(1,14)_ = 8.0, *p* = 0.014; laser stage, *F*_(1.9,26.4)_ = 4.0, *p* = 0.033; virus × laser stage, *F*_(2,28)_ = 5.0, *p* = 0.014; Fig. 5*Eii*). Unlike STN activation, halo-mediated inhibition of STN did not produce a rapid or pronounced change in angular velocity (Fig. 5*F*).

In sum, our results in the open-field assay revealed effects of both STN stimulation and inhibition. Unexpectedly, we found that brief activation of the STN led to a short-lived increase in angular velocity. Importantly, however, on a per mouse basis, the magnitude of this movement did not correlate with the magnitude of deficit in the DMTP, suggesting the two effects are unrelated. On the other hand, sustained STN inhibition led to a modest increase in speed that is consistent with classic models of STN function.

## Discussion

Much work implicates STN activation in suppressing an action in laboratory stop-signal tasks. In these assays a subject is told or trained to stop a specific behavior in response to a specific cue that occurs with some probability within a narrow time window. While such lab-based tasks are extremely useful for modeling behavioral stopping, in the real-world, the signals that stop animal behavior are variable, and often unexpected or surprising. Recent studies have indeed shown that surprising stimuli also can stop ongoing behavior via a fronto-basal-ganglia mechanism; and that surprising events interrupt cognition (Wessel et al., 2016). The current results go further by providing specific neurobiological detail of how surprise-induced activation of STN “stop” circuits also erodes performance in a task requiring cognition.

In our DMTP assay the mouse must maintain a spatial location and action plan across a delay period to obtain a reward. Presumably, this requires maintaining motivation for an objective across time (goal maintenance). Indeed, performance on this task decays with increasing delay, presumably as a consequence of failing to maintain the target location because of distraction or disengagement from the task. When we replaced a “standard” cue that indicated the end of a delay period with a cue that was novel (surprising), this led to a decrement in performance as measured by reduced reward rate, slowed response time on correct responses, and increased error rate, particularly in the form of omission errors.

If surprise decrements cognitive performance by recruiting STN stop circuits, then we reasoned we could mimic the disruptive effects of surprise simply by activating the STN. Indeed, brief optogenetic activation of STN led to a very similar effect as the surprising cue had, reduced reward rate because of increased omission errors and slower response time selectively on correct responses. Further, optogenetic inhibition of STN attenuated the surprise-induced response slowing. However, despite speeding the response time on correct trials, STN inhibition, independent of surprise, led to more errors. Together, these findings lend new support to the hypothesis that changes in STN activity can disrupt cognitive processes related to goal maintenance, working memory, or action planning. It is unclear whether this tendency to omit a response following the surprise cue or STN stimulation is because of a loss of content (i.e., visuospatial information) being held in working memory during the delay period, a loss of task engagement, a cancellation of the response, or some combination of these effects. Regardless, the convergent pattern of effects between surprise and STN activation/inhibition is consistent with the hypothesis that they work via a common mechanism.

The canonical targets of excitatory projection neurons in STN are the SNr and GPi, which are considered the primary output pathways of the basal ganglia. STN activation could thus disrupt an action plan by activating the SNr and GPI, which in turn inhibit thalamo-cortical networks engaged in maintaining a representation of the task. Indeed, evidence supports a predominant role for reciprocal thalamo-frontal projections in sustaining working memory and goal maintenance (Bolkan et al., 2017; Guo et al., 2017; Schmitt et al., 2017). We posit that transient inhibition of these circuits downstream of STN activity could disrupt similar cognitive processes by attenuating recurrent task-related activity in these circuits, resulting in increased errors and slower responding in the DMTP task.

Rather than alleviating the disruptive effects of surprise, STN inhibition reduced performance across both standard-trial and surprise-trial types. This suggests that endogenous STN activity contributes to optimal performance on this task. STN inhibition could interfere with the normal operation of a putative “hold your horses” function that improves performance by delaying action until more information is accrued and appropriately processed into an action plan (Frank, 2006). Indeed, our finding that STN inhibition speeds responding is consistent with this theory. It may also be that optimal performance on the task requires specific spatial and temporal patterns of STN subcircuit activity that simultaneously inhibit some behaviors while permitting others. Other possibilities include that our approach led to incomplete inhibition of STN, or that optogenetic inhibition or excitation of STN induced imbalances in circuit activity that contribute to our observations.

Our results should also be interpreted in light of our findings in the open field assay, which revealed motoric effects of both STN stimulation and inhibition. Using the same brief optogenetic stimulation of the STN as used in the DMTP task, we measured a transient increase in angular velocity in the open field. The simplest interpretation is that this represents a brief involuntary movement. The direction of movement was consistently ipsiversive when light was delivered unilaterally, but similar changes in angular velocity were present when light was delivered bilaterally, though without a directional bias across animals. It is not clear why brief STN activation should trigger an increase in movement, rather than a decrease as predicted by the canonical models. It is possible that transient STN activation briefly increased movement by working through an alternate STN output (e.g., to external segment of globus pallidus), or is a supraphysiologic consequence resulting from synchronous activation of many STN neurons. Notably, a recent study that used sustained optogenetic stimulation over several minutes showed the expected inhibitory effect on ambulatory movement (Guillaumin et al., 2020)

In any case, the finding of STN stimulation elicited movement suggests alternate interpretations of the ChR2 effect in the DMTP, where the performance deficits could be secondary to an involuntary movement. For example, rats use idiosyncratic movements to execute an interval timing task and spontaneous deviations from that movement pattern predicted failures (Gouvêa et al., 2014), the brief movement induced by our STN activation may then interfere with such a strategy. However, several points argue against this possibility. First, we did not detect any within-animal correlation between the change in angular velocity measured in open-field with the change in error rate in the DMTP, suggesting that the two behavioral effects are unrelated. (This despite detecting a within-animal correlation between sensitivity to surprise and sensitivity to ChR2 activation of STN on error rate in DMTP.) Second, the increase in response latency downstream of STN activation was only observed on correct responses, and not on incorrect responses as would be predicted if the slowing were a result of a non-specific motor effect. Further, transient activation of indirect pathway neurons caused mice to disengage (abort) from a sequence task, presumably leading to activation of STN, and this study did not report an involuntary movement (Tecuapetla et al., 2016). Thus, activation of STN directly or through activation of upstream indirect pathway circuits can cause task disengagement, whether or not a movement is elicited. Finally, our bidirectional effects of STN inhibition provide an independent test of our overall hypothesis. However, we cannot strictly rule out that the effects of ChR2 stimulation are secondary to motoric effects.

It may also be the case that the effects of STN activity on interrupting action and cognitive processes cannot be readily decoupled. Thus, it is not currently possible to determine whether effects on cognitive outcomes are secondary to stopping an action versus “independent.” In the human analog to these experiments the effects of surprise on error rate (Wessel et al., 2016) and response speed (Wessel and Aron, 2013) were identified in separate working memory and stimulus-response tasks. Here, we were able to detect both effects in a single assay. Whether response slowing would be seen in humans in a more demanding cognitive task or in mice with a simpler stimulus-response task requires additional testing. One difference between the trial outcome effects seen here and those in humans is that mice tend to omit a response following surprise or STN stimulation whereas humans tend to respond incorrectly. The tendency to omit rather than guess incorrectly may also explain why we did not observe effects of STN activation on incorrect error rate but only on omission error rate. This difference may be accounted for by meta-task cognition that prompts humans to respond with a guess even when a memory has eroded. In both model systems it remains to be determined whether the slower response speed is because of a delayed initiation of movement or slower execution of movement, but a similar mechanism of slowing could further bolster the case for cross-species homology.

In sum, we provide evidence that optogenetic activation or inhibition of STN stop circuits can increase error rate and, respectively, slow or speed responding on a cognitive assay. These findings are consistent with the overall hypothesis that surprise engages STN-based stop circuits to not only slow or stop behavior, but that this also leads to slowing or stopping thought. Future studies may parse whether such effects are mediated by parallel STN subcircuits, or whether any cognitive effects are a secondary/downstream consequence of abruptly stopping a behavior. In either case, the effect may release behavioral and cognitive resources so that the subject can attend to and respond to new or unexpected sensory information in an appropriately adaptive way. This theory also implicates dysfunctional STN stop circuits in cognitive disorders, where excessive motor/behavioral interruptions also interfere with cognitive processes, for example excessive distractibility apparent in attention-deficit hyperactivity disorder.

## Synthesis

Reviewing Editor: Christie Fowler, University of California, Irvine Decisions are customarily a result of the Reviewing Editor and the peer reviewers coming together and discussing their recommendations until a consensus is reached. When revisions are invited, a fact-based synthesis statement explaining their decision and outlining what is needed to prepare a revision will be listed below. The following reviewer(s) agreed to reveal their identity: Melissa Herman, Fu-Ming Zhou.

Both reviewers were positive about the data provided in the manuscript and its relevance to prior studies of the STN in humans. However, several concerns were noted regarding the experimental design and results reported. The reviewers further felt that the authors could address these concerns within 2 months, which would result in significant improvement of the manuscript from its current form.

Reviewer 1:

The current study examines the role of the subthalamic nucleus (STN) in the processing of ‘surprising’ stimuli and the impact of that processing on behavior and cognitive performance, specifically the delayed match to position (DMTP) task. The authors present work testing the hypothesis that surprise activates STN ‘stop’ circuits to slow behavioral responding and impair cognitive performance. Interestingly, the magnitude of perturbation is greater with increased salience of the novel cue but decreased performance is still observed even when the novel cue is less salient. The authors provide evidence that optogenetic activation or inhibition of glutamatergic STN neurons increases error rate and increases or decreases latency to respond, respectively, in the DMTP cognitive assay. The authors also demonstrate that activation or inhibition of STN neurons has motor consequences (increased head angular velocity and increased distance, respectively) as measured in Open Field. Overall, this study is composed of rigorous and well-controlled experiments indicating that the STN is engaged during novel stimuli-induced perturbations in responding during a behavioral task and may play a role in inhibiting behavior to allow the processing of new or surprising information. These data provide important preclinical support for findings observed in human tasks and build on our understanding of the role of STN processing is specific aspects of cognitive performance. There are, however, a few points in the communication of the experimental paradigm and the illustration/communication of the results that should be clarified prior to publication:

Comments:

1. Aspects of behavioral testing should be specified in greater detail. The authors indicate that testing took place over 45 min in the light cycle (Methods, pg 4) but should indicate over what time range and whether or not males and females were running concurrently or in sequence. Authors indicate that “sessions were repeated on consecutive days until we collected at least 25 trials of both laser and non-laser trial per mouse” but a more specific range of sessions and days required to reach criterion should be noted.

2. In the Results section (pg 6) it is stated that the standard cue was replaced with a novel (surprising) cue on 50% of the 3-s delay trials, however Figure 1C appears to indicate that the surprising cue was presented in 25% of trials. This should be clarified/reconciled.

3. It is unclear why the authors chose the 3 s delay, (particularly as the standard rewarded performance was <80%) as opposed to a shorter delay period (e.g. 2 s) that showed better baseline performance with which to compare the ‘surprise’ trials. Some explanation/justification of this experimental parameters should be provided.

4. The mice from the cohort shown in Figure 2 displayed better responding (higher % rewarded) as compared to Fig 1, even though the standard task was identical to that used in Figure 1, this suggests that there may be some variability in performance between cohorts that should be mentioned.

5. The % rewarded appears to be significantly lower in mice from the cohorts shown in Figure 3 and Figure 4 as compared to Figure 1, suggesting that viral injection and/or fiber implant impairs performance. This should be noted.

6. On page 9 of the Results it is noted that “the standard-cue was replaced with the novel-cue (i.e., surprise) in 50% of the 3-s-delay trials and 25% of trials were surprise trials.” This should be clarified. It is also stated that “the laser was activated 50 ms prior to cue presentation, and the laser remained on until the end of the trial” and it should be explicitly stated how long the laser remained on (i.e. the range of laser on times).

7. It would be helpful to see graphs of the % incorrect and % omissions for the optogenetic inhibition experiments as they are communicated for the optogenetic activation (Figure 3E and 3F).

8. On page 11 of the Results, the authors note that “The slowing effect of surprise was reduced by laser in 87% of Halo mice, but only 56% (approximately chance) of eYFP mice.” This aspect of the data would be better communicated by line graphs depicting the within-subject shifts in performance as shown in Figures 1Gi, 2Bi, and 3Gi.

9. It is unclear why the ChR2 and Halo open field testing was performed in new cohorts of mice and not the mice used for testing in Figures 3 and 4. The data would be stronger if the experiments were performed in the same mice. Indeed, as some comparisons and correlations are made in the experimental animals shown in Figures 3 and 4 it is unclear why those data are not included in full.

10. In general, the main effects of all experimental manipulations (surprise, ChR2 activation, Halo inhibition) appear to be concentrated more heavily in the timing of responding, with surprise (Fig 1) and activation of STN (Fig 3) increasing the latency to respond and as a result mainly decreasing the % rewarded by increasing % omissions, and inhibition of STN (Figure 4) blunting the increased latency that occurred with surprise. The % incorrect was relatively unchanged by surprise or STN manipulation, suggesting less that the mice could not perform the test in terms of accuracy, but that they were just slower to perform. Although the authors performed important motor testing of the impact of ChR2 stimulation and Halo inhibition of STN neurons, and did report potential motor confounds, more attention could be paid to the potentially larger role of the STN in stopping action (that then consequentially impairs performance) then by directly impairing cognition, as is suggested in several sections, notably in the Discussion.

Minor comments:

- There is a typo in the legend for Figure 2Bi where it is stated that “...the novel cue elicits a significant decrease in the latency...” when an increase in latency is shown.

- In Figure 3 panels Dii and Eii are missing from the figure legend.

- There appears to be an image missing for Figure 5Bii.

- There is a typo on pg 15 of the Discussion where the word “rule” is repeated in the phrase “rule out rule"

Reviewer 2:

Using optogenetic activation and inhibition of the subthalamic nucleus in mice, this study provides experimental evidence that the subthalamic nucleus can affect cognitive processes. The mechanistic findings in the paper are consistent with the clinical literature that deep brain stimulation of the subthalamic nucleus in humans has multiple non-motor behavioral or cognitive effects, thus advancing our understanding of this crucial brain structure.

I have the following questions for the authors:

1. For optogenetic stimulation of the subthalamic nucleus, unilateral stimulation was used. In theory, unilateral stimulation of the subthalamic nucleus can cause some imbalance between the 2 hemispheres and hence impair the brain function including cognitive function, thus potentially confounding data--please discuss if there is actually no confounding. Was bilateral stimulation tried? If so, what were observed?

2. For optogenetic inhibition of the subthalamic nucleus, bilateral stimulation was used. Why bilateral here? Was unilateral inhibition tried? If so, what were observed?

3. Please provide details of the viral vectors in the method section. The reader will appreciate these details in the current paper.

4. No optic fiber track is visible in Figure 4. The smooth hole in the tissue section in Figure 3 seems to be a blood vessel, not optic fiber-induced lesion. Please explain and discuss.

5. Line 28. The word “deep” is not meaningful here because no nucleus in the basal ganglia can be reasonably described as a superficial nucleus.

## Author Response

We thank the Reviewers f or their thoughtful assessments of our work. We detail our responses point-by-point below. We hope you remain healthy and saf e in these dif ficult times.

Reviewer 1:

The current study examines the role of the subthalamic nucleus (STN) in the processing of ‘surprising’ stimuli and the impact of that processing on behavior and cognitive performance, specifically the delayed match to position (DMTP) task. The authors present work testing the hypothesis that surprise activates STN ‘stop’ circuits to slow behavioral responding and impair cognitive perf ormance. Interestingly, the magnitude of perturbation is greater with increased salience of the novel cue but decreased perfo rmance is still observed even when the novel cue is less salient. The authors provide evidence that optogenetic activation or inhibition of glutamatergic STN neurons increases error rate and increases or decreases latency to respond, respectively, in the DMTP cognitive assay. The authors also demonstrate that activation or inhibition of STN neurons has motor consequences (increased head angular velocity and increased distance, respectively) as measured in Open Field. Overall, this study is composed of rigor ous and well-controlled experiments indicating that the STN is engaged during novel stimuli-induced perturbations in responding during a behavioral task and may play a role in inhibiting behavior to allow the processing of new or surprising information. Th ese data provide important preclinical support f or findings observed in human tasks and build on our understanding of the role of STN processing is specific aspects of cognitive performance. There are, however, a f ew points in

the communication of the experimental paradigm and the illustration/communication of the results that should

be clarif ied prior to publication:

Comments:

1. Aspects of behavioral testing should be specified in greater detail. The authors indicate that testing took place over 45 min in the light cycle (Methods, pg 4) but should indicate over what time range and whether or not males and f emales were running concurrently or in sequence. Authors indicate that “sessions were repeated on consecutive days until we collected at least 25 trial s of both laser and non-laser trial per mouse” but a more specif ic range of sessions and days required to reach criterion should be noted.

Behavioral testing and training began 1-3 hours af ter lights-on and continued until the entire cohort had been run (6-8 hours per day). Male and f emale mice were run concurrently in separate boxes. Sessions were repeated on consecutive days f or 2-13 days until we collected at least 25 trials of each type per mouse . This inf ormation has been added to the methods sectio n on lines 78-81, 132, 143, and 156.

2. In the Results section (pg 6) it is stated that the standard cue was replaced with a novel (surprising) cue on

50% of the 3-s delay trials, however Figure 1C appears to indicate that the surprising cue was presented in

25% of trials. This should be clarif ied/reconciled.

We apologize f or the conf usion. Surprise was presented on 50% of 3-s delay trials, but because only 50% of total trials were 3-s trials (the other trials were 1- or 2-s trials at equal probability), the 3-s surprise trials represented 25% of the total number of trials. This has been clarif ied in the methods lines 130-131 and 149-

150; and is also noted in results lines 197-198.

3. It is unclear why the authors chose the 3 s delay, (particularly as the standard rewarded perf ormance was

<80%) as opposed to a shorter delay period (e.g. 2 s) that showed better baseline performance with which to compare the ‘surprise’ trials. Some explanation/justif ication of this experimental parameters should be provided.

Because we conducted bidirectional manipulations, we chose a delay that resulted in an intermediate level of perf ormance to f acilitate detection of changes in either direction. A note stating this has been added to lines

193-195.

4. The mice f rom the cohort shown in Figure 2 displayed better responding (higher % rewarded) as compared to Fig 1, even though the standard task was identical to that used in Figure 1, this suggests that there may be some variability in perf ormance between cohorts that should be mentioned.

This may ref lect any number of subject or investigator sources of variability and is why all comparisons are made within experiment and within subject whenever possible. One possibility f or this particular dif ference is that in f igure 2, the two cohorts of mice were trained side-by-side for the same amount of time. But because

the B->A mice took somewhat longer to reach criterion, all mice in f igure 2 were trained f or slightly longer periods of time than those in Figure 1. We make note of the variable training lenghts on lines 122-124.

5. The % rewarded appears to be signif icantly lower in mice f rom the cohorts shown in Figure 3 and Figure 4 as compared to Figure 1, suggesting that viral injection and/or f iber implant impairs perf ormance. This should be noted.

Indeed, mice tested with optogenetic manipulations in f igures 3-4 appeared to perform worse than those not tested with optogenetic manipulation in f igures 1-2. However we think this is a consequence of optic f iber tethers that were used to couple the f iber implant to the laser. While coupled, motor performance and task engagement is impacted to some extent. We note that in the untethered training sessions prior to testing with laser these mice perf ormed at levels comparable to those in f igures 1 and a note to this ef f ect has been added on lines 158-162.

6. On page 9 of the Results it is noted that “the standard -cue was replaced with the novel-cue (i.e., surprise) in

50% of the 3-s-delay trials and 25% of trials were surprise trials.” This should be clarif ied. It is also stated that “the laser was activated 50 ms prior to cue presentation, and the laser remained on until the end of the trial” and it should be explicitly stated how long the laser remained on (i.e. the range of laser on times).

We again apologize f or the confusion and this comment is similar to that raised in point #2 above. We have clarif ied in the methods and results the relative f requency of each trial type (lines 130-131, 149-150, and 197-

198. We have also added the range of laser -on times to the portion of the methods section describing the optogenetic inhibition experiments (lines 154-155).

7. It would be helpf ul to see graphs of the % incorrect and % omissions f or the optogenetic inhibition experiments as they are communicated f or the optogenetic activation (Figure 3E and 3 F).

We presume the Reviewer is ref erring to f igure 4. These metrics have now been added as new panels Figure

4E and 4F).

8. On page 11 of the Results, the authors note that “The slowing effect of surprise was reduced by laser in

87% of Halo mice, but only 56% (approximately chance) of eYFP mice.” This aspect of the data would be

better communicated by line graphs depicting t he within-subject shifts in performance as shown in Figures 1Gi,

2Bi, and 3Gi.

We thank the Reviewer f or this suggestion. We now include within-subjects lines connecting individual points to Figures 4Dii, 4Eii, 4Fii, and 4Gii.

9. It is unclear why the ChR2 and Halo open f ield testing was perf ormed in new cohorts of mice and not the mice used f or testing in Figures 3 and 4. The data would be stronger if the experiments were performed in the same mice. Indeed, as some comparisons and correlations are made in the experimental animals shown in Figures 3 and 4 it is unclear why those data are not included in f ull.

We agree on the utility of the within-subjects comparisons. However, the open-field testing was conceived of

as a separate experiment, primarily to quantif y ef fects observed with ChR2 stimulation . Unf ortunately, the mice used in f igure 4 had already been euthanized prior to open-field testing.

10. In general, the main ef f ects of all experimental manipulations (surprise, ChR2 activation, Halo inhibition) appear to be concentrated more heavily in the timing of responding, with surprise (Fig 1) and activation of STN (Fig 3) increasing the latency to respond and as a result mainly decreasing the % rewarded by increasing % omissions, and inhibition of STN (Figure 4) blunting the increased latency that occurred with surprise. The % incorrect was relatively unchanged by surprise or STN manipulation, suggesting less that the mice could not perf orm the test in terms of accuracy, but that they were just slower to perf orm. Although the authors

perf ormed important motor testing of the impact of ChR2 stimulatio n and Halo inhibition of STN neurons, and did report potential motor conf ounds, more attention could be paid to the potentially larger role of the STN in stopping action (that then consequentially impairs perf ormance) then by directly impairing cognition, as is suggested in several sections, notably in the Discussion.

We agree with this point and endeavored to keep our conclusions in line with our dataset e.g., abstract lines

11-12, summary paragraph lines 527-531, as well as the addition on lines 507-510. Our data do not allow us

to claim that ef f ects of STN on cognitive performance are independent of its effects on stopping action. We remain agnostic as to whether it is possible to decouple effects of STN activity on action f rom its here proposed ef f ects on cogntition. Based on currently available data, we argue only that activation of STN ‘stop’ circuits has consequence that are not limited to interrupting action, but also cognitive outcomes.

Minor comments:

- There is a typo in the legend f or Figure 2 Bi where it is stated that “...the novel cue elicits a signif icant decrease in the latency...” when an increase in latency is shown.

This has been corrected on line 652.

- In Figure 3 panels Dii and Eii are missing f rom the f igure legend. All f igure panels are now ref erenced in legends .

- There appears to be an image missing f or Figure 5Bii.

Thank you f or noting this, it was a mistake during editing. The figure f ile was correct and ref erence to Figure

5Bii was removed f rom the text.

- There is a typo on pg 15 of the Discussion where the word “rule” is repeated in the phrase “rule out rule” Thank you. This has been corrected on line 506.

Reviewer 2:

Using optogenetic activation and inhibition of the subthalamic nucleus in mice, this study provides experimental evidence that the subthalamic nucleus can af f ect cognitive processes. The mechanistic f indings in the paper

are consistent with the clinical literature that deep brain stimulation of the subthalamic nucleus in humans has

multiple non-motor behavioral or cognitive effects, thus advancing our understanding of this crucial brain structure. I have the f ollowing questions for the authors:

1. For optogenetic stimulation of the subthalamic nucleus, unilateral stimulation was used. In theory, unilateral stimulation of the subthalamic nucleus can cause some imbalance between the 2 hemispheres and hence impair the brain f unction including cognitive f unction, thus potentially confounding data--please discuss if there is actually no conf ounding. Was bilateral stimulation tried? If so, what were observed?

In general, we use unilateral stimulation based on the principle that activity in one hemisphere is suf ficient f or a behavioral process. No bilateral STN stimulation was attempted. However, this alternative interpretation is now raised in the discussion on lines 467-470.

2. For optogenetic inhibition of the subthalamic nucleus, bilateral stimulation was used. Why bilateral here? Was unilateral inhibition tried? If so, what were observed?

Unilateral inhibition was not assessed. We used bilateral inhibition based on the same principle as above. Because surprise-induced activation of STN even in just one hemisphere would be sufficient to trigger a behavioral disruption, then unilateral inhibition may be insuf f icient to block an ef fect.

3. Please provide details of the viral vectors in the method section. The reader will appreciate these details in the current paper.

This inf ormation has been added at lines 85-90.

4. No optic f iber track is visible in Figure 4. The smooth hole in the tissue section in Figure 3 seems to be a blood vessel, not optic f iber-induced lesion. Please explain and discuss.

We have updated Figures 3A and 4A with new images. We used unsheathed 250 um f ibers and it can sometimes be tricky to identif y the exact implant location in a single 2-d image. When sectioning brains we note which sections contain the lesions sites and make a determination of f iber placement that is sometimes inf ormed by evidence across multiple sections.

5. Line 28. The word “deep” is not meaningf ul here because no nucleus in the basal ganglia can be reasonably described as a superf icial nucleus.

This has been replaced with “ sub-striatal” at line 28.
